# Clinical experience with an online adaptive radiotherapy for prostate cancer: successful treatment time optimization

**DOI:** 10.1186/s12885-026-15768-y

**Published:** 2026-02-19

**Authors:** Hanna Malygina, Bryan Salazar Zuniga, Hendrik Auerbach, Marc Ries, Sven Knobe, Yvonne Dzierma, Jan Palm, Markus Hecht

**Affiliations:** 1 Department of Radiotherapy and Radiation Oncology, Saarland University Medical Center, Kirrberger Str. 100, 66421 Homburg, Germany; 2 Present address: Department of Radiotherapy, Rostock University Medical Center, Rostock, Germany

**Keywords:** Prostate cancer, Online adaptive radiotherapy, Session time, Adaptation time, Varian Ethos, HyperSight, Treatment time, Workflow optimization, In-room time

## Abstract

**Background:**

Online adaptive radiotherapy (oART) can provide dosimetric advantages by accounting for daily anatomic changes, potentially improving target coverage and sparing of organs at risk. However, clinical adoption is sometimes limited by concerns over increased per-session treatment time. In this single-center study, we present 1.5 years of clinical experience focused on reducing the oART session time for prostate cancer patients.

**Methods:**

We analyzed 1366 oART sessions from 69 prostate cancer patients treated on a Varian Ethos system between July 2023 and December 2024. We recorded (i) total session time — time between patient entry and exit from the treatment room, and (ii) adaptation time — time from start of the daily cone-beam-CT acquisition to completion of contour review/correction. We assessed the effects of two time-saving measures: automated contouring of the posterior rectal wall and installation of Varian HyperSight imaging. Statistical comparisons used the Mann–Whitney U test.

**Results:**

Automated posterior rectal wall contouring decreased mean adaptation time from 16.0 to 10.5 min ($p<0.001$). Installation of HyperSight reduced mean total session time from 25.8 to 23.3 min ($p<0.001$); the adaptation component improved by 0.5 min but not statistically significant ($p=0.053$). We achieved a total session time of $\leqslant 30$ min for 93% of sessions.

**Conclusions:**

Cone-beam-CT-guided oART is feasible in routine prostate cancer practice. Our findings indicate that a 30-minute time slot is sufficient for most adaptive prostate cancer treatments, and a median total session time of 23 minutes can be reached through workflow and imaging optimization. Clinics considering oART should note that treatment time decreases with operator experience, and targeted measures can further reduce session duration.

**Supplementary Information:**

The online version contains supplementary material available at 10.1186/s12885-026-15768-y.

## Background

Online adaptive radiotherapy (oART) adapts the daily plan to the patient’s anatomy immediately before treatment, enabling improved target coverage and better sparing of organs at risk compared with non-adaptive approaches. Recent reviews and clinical reports demonstrate dosimetric and potential clinical advantages of oART across multiple sites, which motivates increasing clinical adoption [1–5].

Despite these advantages, some clinics may hesitate to implement oART because of concerns about prolonged per-fraction treatment times and the resulting implications for patient throughput and departmental resources. Practical guides and roadmaps for Ethos implementation emphasize the importance of optimized workflows and staff training to make oART clinically sustainable [6, 7].

Different technical platforms for oART exist. MR-guided systems provide excellent soft-tissue contrast and enable plan adaptation with very detailed anatomy; however, MR-guided adaptive sessions are often longer and reduce throughput compared with CBCT-guided systems, and MR-linacs also require substantially higher capital and operational investments [1, 8, 9]. CBCT-guided oART can deliver many of the dosimetric benefits with generally shorter overall session times than MR-guided solutions.

Recent advances in CBCT hardware and reconstruction (e.g., Varian HyperSight) [10] promise faster acquisitions, a larger field of view, and improved image quality—features that can potentially speed up the adaptation workflow.

In this work, we present our single-center experience (69 prostate cancer patients, 1366 adaptive sessions with the Ethos system (Varian Medical Systems, Palo Alto, CA, USA) [11]) and quantify the impact of two practical measures—automated posterior rectal wall contouring and HyperSight installation—on adaptation and total session time.

## Patients and methods

### Online adaptive workflow and time definitions

A detailed description of the Ethos workflow including some technical details is provided in the Supplementary Material. Here, we summarize the time tracking procedure relevant for the present study.

Total session time is defined as the interval from patient entry to patient exit from the treatment room. This interval comprises patient set-up, imaging, adaptation (contour review/correction), plan recalculation/verification, and delivery. Such a definition was chosen to represent the real duration of an adaptive session, which would help to estimate time slots for the clinical implementation of oART.

Adaptation time begins with the start of the daily CBCT acquisition and ends when contour correction is completed and the adapted plan is ready for calculation. After the CBCT is acquired, a radiation therapist (RTT) inspects image quality and starts AI-based auto-contouring. The responsible physician is contacted via a personal radio device and attends the console to review and, if necessary, correct the contours. Any delay in the physician’s arrival is reflected in the adaptation time.

Time stamps were recorded prospectively to the minute shown on the clock (seconds were not recorded).

### Patient and data selection

Between July 2023 and December 2024, 1977 Ethos sessions in 89 patients were delivered at our center. To obtain a homogeneous cohort, we selected only patients with primary prostate cancer, yielding 69 patients and 1380 sessions. All patients had an ECOG performance score of 0-1, allowing treatments to be delivered entirely on an outpatient basis. The patients were treated with the 2 simultaneous integrated boosts (SIB) concept following an in-house protocol based on the CHHiP trial [12]. They received 20 fractions with the cumulative prescribed doses for the planning target volume, SIB1, and SIB2 being respectively 48 Gy, 57.6 Gy, and 60 Gy.

We excluded sessions where a scheduled (non-adapted) plan was used (n=7), sessions with incomplete time tracking (n=2), and sessions with VMAT (volumetric modulated arc therapy) plans (n=5) because VMAT plan calculation times are atypically long in comparison with IMRT (intensity-modulated radiotherapy) plans and are therefore not used in routine practice at our institution. The final analysis included 1366 sessions from 69 patients (see Table 1 in Supplements.pdf for summary).

### Time-saving measures

#### Automated posterior rectal wall contouring

Our center uses the posterior rectal wall (PRW) as an additional organ at risk to optimize dose distribution (see [3] for rationale). In the Ethos workflow, influencer structures (organs, which can influence the target) are automatically segmented by the system and then verified and, if necessary, corrected by the user. It is also possible to generate derived structures from existing contours, for example by applying margins, creating unions or intersections, or defining inner and outer walls. The PRW, however, is usually obtained by trimming the rectum contour ventrally—a derivation not directly supported by the system. As a result, the PRW was the only contour that had to be created entirely manually, which substantially prolonged adaptation time.

From session 215 onward, we implemented an automated algorithm to generate the PRW from the rectum contour by: (1) expanding the rectum contour anterior–posteriorly by 0.2 cm and laterally by 5 cm to smooth edges; (2) shrinking the helper structure posteriorly by 1.3 cm (empirically chosen); and (3) cropping the original rectum with this helper structure to yield the posterior wall (for a detailed description and figures, see [13]). This automation removed the need for manual PRW contouring and thus provided a clear reduction in adaptation time.

#### HyperSight installation

From session 1059 onward, we upgraded our on-board imaging to Varian HyperSight [10]. HyperSight offers faster CBCT acquisition, an expanded field of view, and improved contrast/artifact behavior while providing Hounsfield units accuracy suitable for dose calculations. The faster acquisition and improved image quality can shorten adaptation and pre-treatment imaging times, although advanced reconstruction algorithms may change reconstruction latency.

We note that Ethos 2.0—with on-CBCT native plan recalculation without CBCT-to-CT deformable registration—was not available at our site during the study. Therefore, potential effects from that workflow were not assessed.

### Data filtering and statistical analysis

To estimate the isolated effect of each measure, we applied specific filters:

#### Influence of PRW contouring automation

To quantify the effect of automated PRW contouring, we compared adaptation times between sessions with manual and automated PRW delineation. Since five of six physicians began treating while PRW contours were still generated manually, we excluded the first 30 adapted sessions for each of these physicians to reduce bias from individual learning processes—a pragmatic definition of the learning phase, which roughly corresponds to one week of Ethos operation (6 patients/day). The analysis was further restricted to sessions before the HyperSight installation (the 6th physician started after the installation of the HyperSight, so their learning phase does not affect this comparison). The filtering is summarized in Table 2 in Supplements.pdf.

#### Influence of HyperSight

To estimate HyperSight’s effect, we excluded sessions with manual PRW contouring and the initial learning phases described above for all 6 physicians. This isolates HyperSight’s contribution to adaptation and total session time. The filtering is summarized in Table 2 in Supplements.pdf.

#### Statistical Analysis

The statistical significance of both time-saving measures was assessed using two-sided Mann–Whitney U tests, with effect sizes estimated using Cohen’s *r* for non-parametric data (as described in [14]). A *p*-value $<0.05$ was considered indicative of statistical significance. Absolute *p*-values are reported, and no Bonferroni correction was applied due to the limited number of independent comparisons. All analyses were conducted using custom Python scripts with the NumPy, SciPy, and statistics libraries.

## Results

### Patient characteristics

In total, 69 patients (Table 1) with confirmed prostate cancer were included in the analysis. Tumor staging indicated that 40 patients (58%) presented with T1 disease, 28 (41%) with T2, and 1 patient (1%) with T3. Androgen deprivation therapy was prescribed for 26 patients, guided by clinical criteria and risk classification. Adaptive radiotherapy was delivered predominantly using IMRT, most commonly with 9-beam or 12-beam configurations. In four cases, IMRT beam arrangements varied across treatment sessions, while two patients alternated between IMRT and VMAT.

**Table 1:** Demographic characteristics

	mean	min - max
Age, years	72.2	55 - 83
NCCN risk group	# of patients	% of patients
Low	22	31.9
Intermediate	43	62.3
High	4	5.8
Gleason (ISUP) grade	# of patients	% of patients
6 (1)	17	24.6
7a (2)	36	52.2
7b (3)	12	17.4
8 (4)	3	4.3
Cancer stage	# of patients	% of patients
T1a	2	2.9
T1b	1	1.5
T1c	37	53.6
T2a	3	4.4
T2b	3	4.4
T2c	22	31.9
T3	1	1.5
Androgen	# of patients	% of patients
deprivation	26	37.7
therapy		
	mean	min - max
iPSA, ng/ml	7.2	0.08 - 27
Plan modality	# of patients	% of patients
IMRT 09	23	33.3
IMRT 12	40	58.0
Combination	6	8.7

Demographic characteristics include age, NCCN (National Comprehensive Cancer Network) risk group, Gleason and ISUP (International Society of Urological Pathology) grades, cancer stage, use of androgen deprivation therapy, the latest iPSA value before or shortly after the start of the treatment, as well as the plan modality. Some patients received different plan modalities in different sessions (represented by “Combination”)

**Fig. 1:**
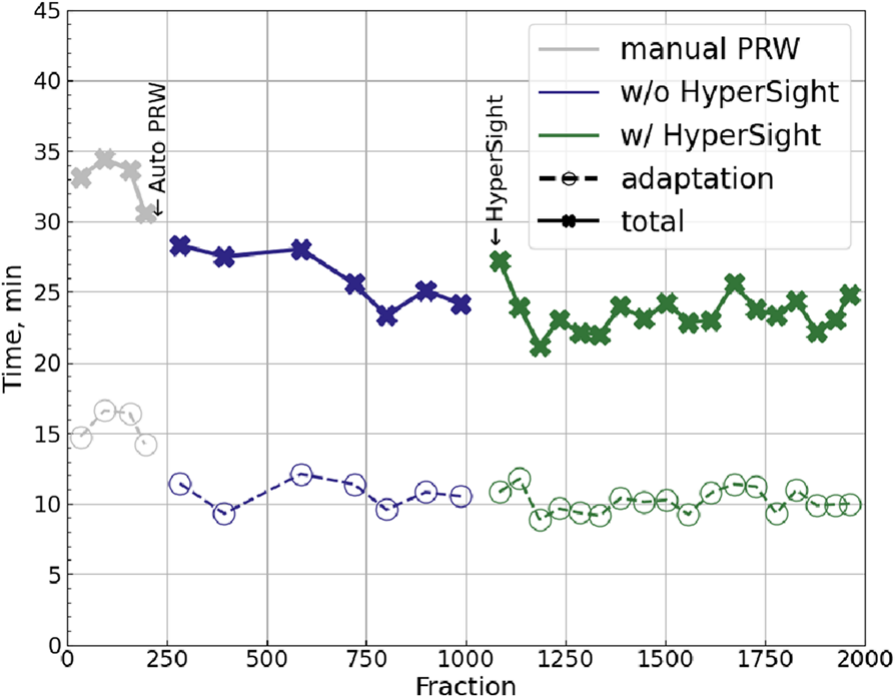
Chronological trends of total session time (crosses and solid lines) and adaptation time (circles and dashed lines). Each point represents the mean of 50 (or fewer) consecutive sessions. The time points at which the two time-saving measures were introduced—automated PRW contouring and the implementation of HyperSight—are indicated schematically with arrows. Gray points correspond to manual PRW contouring without HyperSight, blue-violet points to automated PRW contouring without HyperSight, and green points to automated PRW contouring with HyperSight

The distribution of plan modalities used at our clinic varied only marginally between the compared fraction groups. During the initial phase of Ethos implementation, when manual PRW contouring was employed and HyperSight was not yet available (this period of time corresponds to the gray points on Fig. 1), 66.5% of all fractions were delivered using 12-beam IMRT. For fractions treated with automated PRW contouring but without HyperSight (the blue-violet points on Fig. 1), 12-beam IMRT was used in 64.6% of cases. Following the installation of HyperSight (the green points on Fig. 1), this proportion was 63.8%.

### Total session and adaptation time

**Fig. 2:**
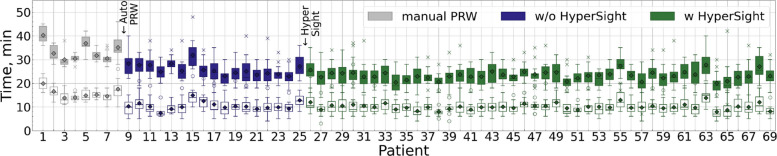
Total session time (filled boxes) and adaptation time (empty boxes) in chronological order by patient. Each boxplot represents one patient. The time points at which the two time-saving measures were introduced—automated PRW contouring and the implementation of HyperSight—are indicated schematically with arrows. Gray indicates manual PRW contouring without HyperSight, blue-violet indicates automated PRW contouring without HyperSight, and green indicates automated PRW contouring with HyperSight. Boxes show the interquartile range (Q1–Q3), the horizontal line marks the median, and diamonds indicate the mean. Outliers are shown as dots for adaptation time (empty boxes) and crosses for total session time (filled boxes), defined as values outside the interval [Q1 – 1.5IQR, Q3 + 1.5IQR], where IQR is the interquartile range

Figure 1 illustrates the chronological trends in adaptation and total session time (no data filtering applied). Each data point represents the mean of up to 50 consecutive sessions, averaged in non-overlapping blocks that correspond to distinct workflow phases: manual PRW contouring (gray points), automated PRW contouring without HyperSight (blue-violet points), and automated PRW contouring with HyperSight (green points). Arrows in Fig. 1 indicate the introduction of automated PRW contouring (session 215) and HyperSight (session 1059). A complementary patient-sorted boxplot representation of the same sessions is provided in Fig. 2. The patients are sorted chronologically.

All IMRT plans were analyzed together, as total treatment time was generally not influenced by the number of beams. As an illustrative example, we examined fractions treated after HyperSight installation—the largest subgroup of sessions—and observed no significant difference in total treatment time between 9- and 12-beam IMRT plans (mean difference 0.3 min, Mann-Whitney U test resulted in $p=0.23$).

**Fig. 3:**
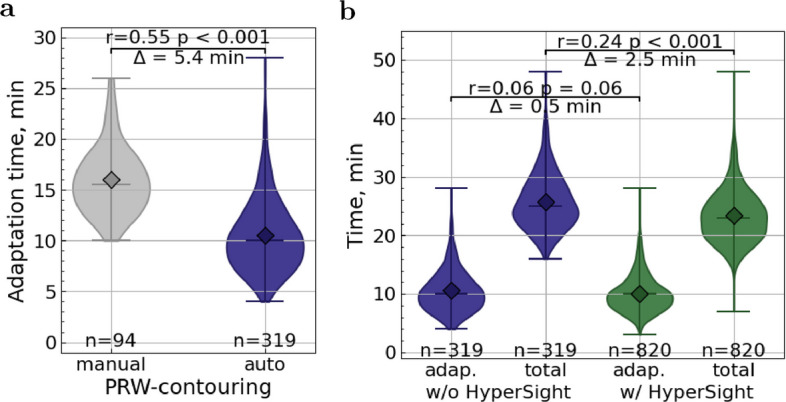
Influence of time-saving measures. **a** Adaptation time with manual vs. automated (auto) PRW contouring. **b** Adaptation and total session time without and with HyperSight. In **a** and **b**, the mean difference ($\Delta$), the Cohen’s *r* effect size, and the Mann–Whitney *p*-value are shown. Horizontal lines indicate the maximum, the median, and the minimum, diamonds mark the mean

Influence of automated PRW contouring. Automated PRW contouring significantly reduced adaptation time, with the mean decreasing from 16.0 to 10.5 min and the median from 15.5 to 10.0 min ($p<0.001$, $r = 0.55$ indicating a large effect size), when applying the data filtering described above (Fig. 3a).

Influence of HyperSight. Installation of HyperSight reduced the total session time, with the mean dropping from 25.8 to 23.3 min and the median from 25.0 to 23.0 min ($p<0.001$, with $r=0.24$ indicating a small-to-medium effect). The adaptation component showed a small, non-statistically significant improvement, with the mean decreasing from 10.55 to 10.06 min and the median remaining at 10 min ($p=0.053$, $r=0.06$ suggesting a minimal effect) (Fig. 3b).

The combined implementation of automated PRW contouring and HyperSight installation achieved 93% of sessions lasting $\leqslant 30$ minutes and 22% lasting $\leqslant 20$ minutes. The mean total session time across post-intervention sessions was 23.3 min (95% CI [23.0, 23.7]), and the mean adaptation time was 10.1 min (95% CI [9.8, 10.3]).

## Discussion

Fast workflows are essential to preserve the benefits of oART, as longer intervals between CBCT scans may increase the likelihood of intra-fractional changes [15] that may compromise the dosimetric benefits [3, 16].

Our findings demonstrated that practical workflow modifications—automated contouring and improved CBCT imaging (HyperSight)—can meaningfully shorten adaptation and total session times in CBCT-guided oART for prostate cancer. We acknowledge that the posterior rectal wall is a center-specific structure that is not universally used, and therefore this particular time-saving measure may not be directly applicable at other institutions. However, our results demonstrate the general potential of automated contouring to reduce adaptation time in online adaptive workflows.

Conversely, our early experience with VMAT plans confirmed that they substantially prolong session times due to longer optimization and calculation, while providing only marginal dosimetric gains compared to IMRT. For this reason, VMAT was ultimately excluded from routine adaptive treatments at our center, and all time analyses presented here are based on IMRT plans.

Our results are consistent with other recent Ethos-based clinical series reporting median total session times in the range of 20–35 minutes and adaptation components around 15 min, depending on definition, disease site, and local workflow specifics [2, 6, 7, 15–20].

For instance, in [17], the authors reported median total session times (patient open to patient close) of about 25 min and median adaptation times (first to second CBCT) of about 15 min for 198 sessions from prostate bed patients. Another analysis [18] reported somewhat longer clinical treatment times (patient open to patient close: 34 ± 6 min) for 184 sessions from prostate patients in early Ethos experience.

Our mean adaptation time of 10.54 min is comparable to the 9.5 min reported by Stanley et al. [6], who analyzed about 1000 adaptive sessions (mixed disease sites) without HyperSight. Their total session time of 34.5 min is notably longer than our pre-HyperSight mean (25.8 min), likely reflecting the heterogeneity of disease sites, which can influence the session time, as was shown in [2, 19, 20].

Some centers have optimized adaptive workflow by redefining staff roles. For example, in [7], trained RTT review the AI-generated contours of influencers, with physicians verifying only the target contours. This workflow yielded a median total session time (in-room time) of 24 min, similar to our 23 min median with HyperSight. Most sessions (92.2%) in that study also used HyperSight CBCT. Another center implemented an RTT-only adaptive workflow for the bladder cancer patients [21], saving on average 3.5 min per session, despite 16% of sessions requiring escalation to a physician or physicist, which could prolong sessions.

We also observed substantial patient-specific variability even under standardized conditions (automated PRW contouring and HyperSight). Some patients required longer treatment times (e.g., patients #55, 63, and 68 averaged 27.3, 27.7, and 27.1 min, respectively, Fig. 2), while others required considerably less time (e.g., patients #34 and 64 averaged 20.4 and 20.0 min, Fig. 2). A similar variability is evident in [7]. Since 93% of our sessions were completed within 30 minutes, a 30-minute time slot seems adequate for most adaptive sessions, while still allowing flexibility for patients who require longer.

Interestingly, another oART system, uRT-linac 506c (Shanghai United Imaging Healthcare Co., Ltd, Shanghai, China), which is based on fan-beam-CT, has demonstrated comparable time performance. In [22], ten patients with cervical cancer (278 adapted fractions) were treated with this system. The mean total session time—from the initial image acquisition until the end of the treatment delivery—was 22.8 min, auto-segmentation and review of regions-of-interest required 10.14 min, and plan generation and evaluation 5.03 min. The results are in close agreement with a similar study using Ethos [23], in which 27 patients (756 fractions) with cervical cancer were analyzed. The authors reported mean total treatment time of 22.9 min (defined in the same way as in [22]), with mean time of 11.4 min for auto-contouring and contour editing, and 4.6 min for plan creation and selection. Together, these findings indicate that currently available oART systems achieve similar performance and may already be operating near the practical limits of today’s technology.

Previous studies [6, 18, 19, 24] have emphasized that imaging quality, AI-contouring quality, staff roles, and training are the primary determinants of throughput and should be optimized to make oART feasible in routine practice. CBCT-guided oART particularly benefits from high-quality CBCT and robust, efficient auto-segmentation, as contour verification and correction are critical drivers of adaptation time [7, 18].

Regarding HyperSight, vendor documentation highlights its 6 s CBCT acquisitions, larger field-of-view, and improved soft-tissue contrast—features that can shorten acquisition time and reduce the need for repeated scans due to artifacts or field-of-view limitations. The observed decrease in total session time after HyperSight installation likely reflects a combination of faster CBCT acquisition, improved image usability (reducing re-acquisitions and contour edits), and increasing staff experience. Note that faster acquisition alone does not guarantee shorter overall session times if reconstruction latency is high; however, in our implementation, the net effect was a meaningful reduction in total session duration.

Strengths. This is a large real-world dataset: more than 1300 adaptive sessions in 69 prostate cancer patients. We focused on the in-room time and the adaptation time—metrics directly relevant for departmental scheduling and clinical feasibility. 

Limitations. This is a single-center, retrospective analysis with manual time stamps (minute resolution) and pragmatic filters for learning phases. This study focused only on prostate cancer; findings may not directly generalize to other disease sites where contouring is more complex. Patient throughput also depends on departmental staffing and scheduling, which were not analyzed here. The influence of patient mobility on total treatment time was not assessed, as all patients included in this study had comparable and good mobility. Consequently, centers treating patients with reduced mobility on Ethos may experience longer total session times than reported here.

## Conclusion

CBCT-guided oART for prostate cancer is feasible and deliverable with clinically acceptable session times. Treatment time decreases with operator experience and targeted interventions: automating repetitive contouring tasks (e.g., posterior rectal wall) and upgrading CBCT acquisition/reconstruction (HyperSight) led to measurable reductions in adaptation and total session durations in our series. After optimization, the median total session (in-room) time was 23 min, with 93% of fractions completed in $\leqslant 30$ min. Clinics considering oART should balance expected dosimetric advantages against initial time investments, plan for staff training/role definition, and evaluate targeted optimization that can make adaptive workflows efficient and sustainable. Future studies should evaluate whether similar workflow optimizations can be generalized across disease sites and institutions.

## Supplementary Information

Supplementary Material 1.

## Data Availability

The datasets analyzed during the current study are not publicly available but could be shared by the corresponding author on reasonable request.

## Abbreviations

oART: Online adaptive radiotherapy
CBCT: Cone-beam computed tomography scan
IMRT: Intensity-modulated radiotherapy
VMAT: Volumetric-modulated arc therapy
PRW: Posterior rectal wall
AI: Artificial intelligence
Q1, Q3: Quartile 1, quartile 3
IQR: Inter-quartile range
RTT: Radiation therapist
