# Supplementary material for: Clinical experience with an online adaptive radiotherapy for prostate cancer: successful treatment time optimization
Source: BMC Cancer. 2026 Feb 19;26:301. doi: 10.1186/s12885-026-15768-y (PMC12930564; doi:10.1186/s12885-026-15768-y)
Supplement: Supplementary file 1 — Supplementary Material 1. [file 12885_2026_15768_MOESM1_ESM.pdf]

Clinical experience with an online adaptive radiotherapy for prostate cancer: successful treatment time optimization

1 Supplement: Data selection

Table 1: Data selection

Step	Number of sessions (patients)
All Ethos sessions delivered (Jul 2023 – Dec 2024)	1977 (89)
Selected: primary prostate cancer only	1380 (69)
Excluded: scheduled (non-adaptive) plans	–7
Excluded: incomplete time tracking	–2
Excluded: VMAT plans (atypically long calculation times)	–5
Final dataset for analysis	1366 (69)

Table 2: Data filtering

	Sub-analysis	
Filter	Manual vs. automated PRW contouring	Without vs. with HyperSight
Learning phase	exclude	exclude
Sessions with HyperSight	exclude	include
Sessions without HyperSight	include	include
Manual PRW contouring	include	exclude

2 Supplement: Ethos online-adaptive workflow

The CBCT-based online adaptive radiotherapy (ART) workflow implemented on the Varian Ethos system follows a structured process that builds upon conventional external beam radiotherapy planning while enabling daily plan adaptation based on the patient's anatomy of the day.

As in standard percutaneous radiotherapy, treatment begins with the acquisition of a planning CT. On this dataset, the clinical target volume (CTV), planning target

volume (PTV), and organs at risk (OARs) are delineated, and the dose prescription is defined.

Based on the planning CT, a treatment intent is created. In the Ethos system, the treatment intent comprises a predefined planning template that specifies the treatment technique and optimization objectives. The user selects one of the available predefined delivery scenarios, including intensity-modulated radiotherapy (IMRT) with 7, 9, or 12 equidistant static beams, or volumetric modulated arc therapy (VMAT) with 2 or 3 arcs. The treatment intent serves as the reference for all subsequent adaptive fractions.

At our clinic, three IMRT plans with different beam numbers are routinely generated for each patient, and the clinically preferred plan is selected and approved for treatment. During the initial phase of Ethos implementation at our institution, VMAT plans were also created and occasionally used for treatment. However, due to the substantially longer online optimization and calculation times associated with VMAT, this technique was later discontinued in routine clinical practice. Fractions delivered with VMAT were therefore excluded from the present study.

Once approved, the selected plan defines the treatment intent and is used as the template for all adaptive treatment sessions. During each adaptive session, only the technique specified in the active treatment intent is used. In certain situations, a revision of the treatment intent may be required. This can occur, for example, when prescription or fractionation is modified, or if OAR constraints cannot be met on-couch with the current planning directives, requiring alternative objective prioritization or additional help-structures. A revision may be performed with or without acquisition of a new planning CT and can result in the selection of a different treatment technique. The revised treatment intent is then used for all subsequent fractions.

For each treatment fraction, the patient is first positioned on the treatment couch, after which a cone-beam CT (CBCT) is acquired to capture the daily anatomy. Following verification of image quality and image registration, the system propagates the contours from the planning CT to the CBCT using deformable image registration. These propagated contours are reviewed and, if necessary, manually adjusted by the user. Based on the anatomy of the day, the Ethos system automatically generates two dose distributions for clinical comparison:

1. Scheduled plan: the dose distribution that would result from delivering the original reference plan (defined by the treatment intent) to the daily anatomy without re-optimization.
2. Adapted plan: a newly optimized treatment plan that aims to reproduce the original planning objectives of the treatment intent while accounting for the current anatomy.

Both plans are evaluated online with respect to target coverage and OAR dose constraints. The treating physician selects the clinically preferred plan and approves it for delivery.

Following plan selection, the approved plan is automatically exported to the Mobius3D system (Varian Medical Systems) for independent patient-specific quality assurance (QA). Mobius3D performs a secondary dose calculation based on an independent beam model and compares the calculated dose distribution and dose–volume

metrics against the treatment planning system results. A medical physicist reviews the QA results and approves the plan prior to treatment.

Before irradiation, a final verification CBCT may optionally be acquired to confirm that the patient position has remained stable since plan approval. If required, a three-dimensional translational couch correction can be applied. However, no further contour adaptation or plan re-optimization is possible after acquisition of this second CBCT.

3 Supplement: Technical details

The cranio-caudal extension of the planning CT scan varied individually among patients and depended on patient anatomy; L4 was required to be fully visible on the scan. This resulted in a field of view of approximately 38 ± 2cm in the cranio-caudal direction, with interpatient variability but without any clear time-dependent trend.

- Without Hypersight: CBCT Mode Pelvis (an energy of 125 kV, an exposure of 1074 mAs, a scan time of 36.7 s), exposure Auto, no extended CBCT, Reconstruction Mode THREE_D, Filter standard, Ring Suppression MEDIUM.
- With HyperSight: CBCT Mode Pelvis (an energy of 125 kV, an exposure of 469 mAs, a scan time of 5.9 s), exposure Auto, no extended CBCT, Reconstruction mode: iCBCT Acuros (or iCBCT MAR if a patient has any metallic objects).
